# Supplementary material for: Hepatobiliary phase signal intensity: A potential method of diagnosing HCC with atypical imaging features among LR-M observations
Source: PLoS One. 2021 Sep 13;16(9):e0257308. doi: 10.1371/journal.pone.0257308 (PMC8437291; doi:10.1371/journal.pone.0257308)
Supplement: S1 Text — (DOCX) [file pone.0257308.s001.docx]

**S1 Text. MRI sequences included in liver dynamic MRI:**

Dynamic liver MRI included T1 weighted 3D gradient-echo imaging was obtained prior to contrast injection and in the arterial phase (18s delay from the time of aortic enhancement using bolus tracking method), portal venous phase (60s), delayed phase (90s) transitional phase (150s), and hepatobiliary phase (20min) after contrast agent injection.

Other MRI sequences included an axial dual-echo T1 weighted breath-hold gradient echo sequence for acquisition of in-phase and out-of-phase images, an axial respiratory-triggered turbo spin-echo T2 weighted sequence with fat saturation, an axial half-Fourier acquisition single-shot turbo spin-echo T2 weighted sequence with fat saturation, and diffusion weighted imaging with respiratory-triggered single-shot echo planar imaging sequences with *b* values of 0, 50, 400 and 800 sec/mm2 or 50, 400 and 800 sec/mm^2^.
