# Supplementary material for: Hepatobiliary phase signal intensity: A potential method of diagnosing HCC with atypical imaging features among LR-M observations
Source: PLoS One. 2021 Sep 13;16(9):e0257308. doi: 10.1371/journal.pone.0257308 (PMC8437291; doi:10.1371/journal.pone.0257308)
Supplement: S1 Table — (DOCX) [file pone.0257308.s002.docx]

| **S1 Table.** Sensitivity, specificity, positive predictive value (PPV), negative predictive value and accuracy for HCC based on LI-RADS v2018 of eligible hepatic observations | | | | | | |
| --- | --- | --- | --- | --- | --- | --- |
|  | **Sensitivity (%)** | **Specificity (%)** | **PPV (%)** | **NPV (%)** | **Accuracy (%)** | ***P*-value** |
| LR-4 and LR-5 combined | 92.9  (710/764)  [90.9, 94.7] | 94.9  (497/524)  [92.6, 96.6] | 96.3  (710/737)  [94.8, 97.4] | 90.2  (497/551)  [87.7, 92.3] | 93.7  (1207/1288)  [92.2, 95.0] | <0.001 |
| LR-5 | 71.6  (547/764)  [68.3, 74.8] | 98.3  (515/524)  [97.8, 99.2] | 98.4  (547/556)  [97.0, 99.2] | 70.4  (515/732)  [67.9, 72.7] | 82.5  (1062/1288)  [80.3, 84.5] | <0.001 |
| *Sensitivity, specificity, PPV, NPV and accuracy for LR-M is calculated for non-HCC malignancy not HCC.  HCC, hepatocellular carcinoma; LI-RADS, Liver Imaging-Reporting and Data System; PPV, positive predictive value; NPV, negative predictive value | | | | | | |
