# Supplementary material for: Hepatobiliary phase signal intensity: A potential method of diagnosing HCC with atypical imaging features among LR-M observations
Source: PLoS One. 2021 Sep 13;16(9):e0257308. doi: 10.1371/journal.pone.0257308 (PMC8437291; doi:10.1371/journal.pone.0257308)
Supplement: S2 Table — (DOCX) [file pone.0257308.s003.docx]

| **S2 Table.** *X*^2^-test result HCC vs. dark signal intensity group in hepatobiliary phase | | | |
| --- | --- | --- | --- |
|  | Low, and iso-to-high SI | Dark SI | *P*-value |
| Non-HCC LR-M (n=64) | 44 | 20 | **0.030** |
| HCC LR-M (n=42) | 20 | 22 |  |
| HCC, hepatocellular carcinoma; SI, signal intensity. | | | |
