# Supplementary material for: Hepatobiliary phase signal intensity: A potential method of diagnosing HCC with atypical imaging features among LR-M observations
Source: PLoS One. 2021 Sep 13;16(9):e0257308. doi: 10.1371/journal.pone.0257308 (PMC8437291; doi:10.1371/journal.pone.0257308)
Supplement: S3 Table — (DOCX) [file pone.0257308.s004.docx]

| **S3 Table.**. *X*^2^-test result iCCA or cHCC-CCA vs. low signal intensity group in hepatobiliary phase | | | |
| --- | --- | --- | --- |
|  | Dark, and iso-to-high SI | Low SI | *P*-value |
| Rest of LR-M (n=45) | 27 | 18 | **0.002** |
| iCCA or cHCC-CCA (n=61) | 18 | 43 |  |
| iCCA, intrahepatic mass-forming cholangiocarcinoma; cHCC-CCA, combined hepatocellular-cholangiocarcinoma; SI, signal intensity. | | | |
