# Supplementary material for: Hepatobiliary phase signal intensity: A potential method of diagnosing HCC with atypical imaging features among LR-M observations
Source: PLoS One. 2021 Sep 13;16(9):e0257308. doi: 10.1371/journal.pone.0257308 (PMC8437291; doi:10.1371/journal.pone.0257308)
Supplement: Attachment — Submitted filename: Response to Reviewers.docx [file pone.0257308.s006.docx]

**Review Comments to the Author**

Please use the space provided to explain your answers to the questions above. You may also include additional comments for the author, including concerns about dual publication, research ethics, or publication ethics. (Please upload your review as an attachment if it exceeds 20,000 characters)

**Reviewer #1:** This paper aims to assess whether hepatobiliary phase signal intensity can be used to differentiate HCC and non-HCC malignancies within LR-M observations.

The study looks original even though a larger sample could have made significant data for HCC with iso-to-high signal.

**Comment #1** - English needs to be revised: there are many typos (for example, lines 97, 193 and 267)

🡪 We are sorry for the typos. We asked a native English speaker to review our revised manuscript.

**Introduction**

**Comment #2**. -“the diagnostic performance of LR-M for non-HCC malignancy has been variable” This is too vague. Please provide %

🡪 As suggested by Reviewer #1, we included the range of reported sensitivity and specificity of LR-M features and revised our sentence to read as follows: “..the diagnostic performance of LR-M features for non-HCC malignancy has been variable with reported sensitivity of 9-83% and specificity of 69-97%” (reference: Kim YY, Kim MJ, Kim EH, Roh YH, An C. Hepatocellular Carcinoma versus Other Hepatic Malignancy in Cirrhosis: Performance of LI-RADS Version 2018. Radiology. 2019;291(1):72-80).

**Comment #3** - I suggest you to discuss here or in the discussion section about these recent papers to highlight that the same clinical issue has been recently investigated in different ways (“Recently, some studies …..[”Oestmann et al, Jiang H et al”]) including evaluation or serum markers and deep learning, but that to your knowledge no prior study performed a quantitative assessment on HBP.

Oestmann PM, Wang CJ, Savic LJ, Hamm CA, Stark S, Schobert I, Gebauer B, Schlachter T, Lin M, Weinreb JC, Batra R, Mulligan D, Zhang X, Duncan JS, Chapiro J. Deep learning-assisted differentiation of pathologically proven atypical and typical hepatocellular carcinoma (HCC) versus non-HCC on contrast-enhanced MRI of the liver. Eur Radiol. 2021 Jan 6. doi: 10.1007/s00330-020-07559-1.

Jiang H, Song B, Qin Y, Chen J, Xiao D, Ha HI, Liu X, Oloruntoba-Sanders O, Erkanli A, Muir AJ, Bashir MR. Diagnosis of LI-RADS M lesions on gadoxetate-enhanced MRI: identifying cholangiocarcinoma-containing tumor with serum markers and imaging features. Eur Radiol. 2021 Jun;31(6):3638-3648. doi: 10.1007/s00330-020-07488-z. Epub 2020 Nov 27. PMID: 33245494.

🡪 As suggested by Reviewer #1, we included the above references and discussed them in the introduction as follows: “To our knowledge, while there have been studies analyzing tumor serum markers, imaging findings and deep learning (15, 16), no prior study has performed a quantitative assessment of hepatobiliary phase signal intensity in order to differentiate a LR-M observation.”

**Comment #4** - You investigated this topic just on gadoxetate-disodium MRI. I would suggest to add a paragraph on why this contrast agent is often used in clinical practice in cirrhotic population. Please keep in mind that what happens in Eastern and Western countries may not be similar. As such, I would encourage to write a specific sentence highlighting the benefit of this contrast agent in this cohort of patients (see references

Zech CJ, Ba-Ssalamah A, Berg T, Chandarana H, Chau GY, Grazioli L, Kim MJ, Lee JM, Merkle EM, Murakami T, Ricke J, B Sirlin C, Song B, Taouli B, Yoshimitsu K, Koh DM. Consensus report from the 8th International Forum for Liver Magnetic Resonance Imaging. Eur Radiol. 2020 Jan;30(1):370-382. as specific guidelines

Vernuccio F, et al. AJR Am J Roentgenol. 2019 Aug;213(2):W57-W65. doi: 10.2214/AJR.18.20979. for its role in the diagnostic performance in indeterminate lesions

🡪 As suggested by Reviewer #1, we added sentences to provide basis for why we analyzed gadoxetic acid- enhanced MRI and also included the above references.

**Materials and methods**

Study cohort

**Comment #5** - you included “patients with underlying liver cirrhosis or chronic B-viral hepatitis”. Please consider that LI-RADS includes also patients with prior HCC. Please be consistent with LI-RADS criteria

🡪 We agree with Reviewer #1 that patients with prior HCC can be also evaluated using LI-RADS criteria. However, we chose to evaluate treatment-naive patients with chronic hepatitis B virus infection and/or liver cirrhosis similar to other previous published studies; yet, we made sure to acknowledge this under limitations.

- Rhee H, Cho ES, Nahm JH, Jang M, Chung YE, Baek SE, et al. Gadoxetic acid-enhanced MRI of macrotrabecular-massive hepatocellular carcinoma and its prognostic implications. **Journal of hepatology**. 2021;74(1):109-21.

- An C, Kim DW, Park YN, Chung YE, Rhee H, Kim MJ. Single Hepatocellular Carcinoma: Preoperative MR Imaging to Predict Early Recurrence after Curative Resection. **Radiology**. 2015;276(2):433-43.

- Kim YY, Kim MJ, Kim EH, Roh YH, An C. Hepatocellular Carcinoma versus Other Hepatic Malignancy in Cirrhosis: Performance of LI-RADS Version 2018. **Radiology**. 2019;291(1):72-80.

**Comment #6**- “T1 weighted 3D gradient-echo hepatobiliary phase (HBP) was obtained 20 minutes after contrast agent injection." Has the hepatobiliary phase been judged appropriate by a radiologist for all examinations? If so, specify it. Indeed, Image F of Figure 2 is not an adequate hepatobiliary phase, which compromises signal intensity assessment. Please see these criteria in this paper and mention it: Khouri Chalouhi C, et al. Eur Radiol. 2019 Jun;29(6):3090-3099.

- Did you use 10 ml of gadoxetate-disodium regardless of the patient's weight?

🡪 Yes, while the hepatobiliary phase image was not judged at the time of the image acquisition, image qualities were judged at the time of MRI reading and if not diagnostic, we repeated the exam (image acquisition) in daily practice. Since we performed a retrospective study, all the images were judged at the timing of MRI reading and if inadequate, exam was repeated; thus we believe an adequate quality control was done. We agree that Figure 2 does not appear as a typical HBP image. Sometimes, even when the patient does not have a biliary obstruction, HBP image can appear as Figure 2 even after repeat examination. Since we use both extracellular agent (ECA) and EOB contrast at our hospital, we also confirmed the type of contrast used and the timing of image aquisition since contrast injection. We can assure that the Figure 2 patient underwent EOB contrast MRI and the contrast is pooled in bile ducts in other axial images along z-axis.

As proof, below are the other HBP images of Figure 2 patient, and you can see that while the signal intensity of intrahepatic vessels are not lower than that of liver parenchyme, these images are indeed EOB contrast images since the contrast is pooled in gall bladder and bile ducts. These images were taken abour 20 minutes past time of contrast injection.

This same patient also underwent EOB contrast MRI after surgery (left lobectomy) and the HBP images were as follows:

Similar to pre-operative EOB contrast MRI, the intrahepatic vessels did not show lower signal intensity than liver parenchyme in HBP, but the HBP was obtained when contrast is pooled in the biliary ducts.

🡪 Yes, we used 10mL of gadeoxetic-disodium regardless of patient’s weight as it is our hospital protocol

**Results**

**Comment #7** - You should not emphasize or specifically focus on the 3 HCC with iso-to-high signal as this does not represent a relevant data (too few patients), rather more centrality should be given to the other significative data obtained.

🡪 We thank Reviewer #1 for his or her comment. We agree with Reviewer #1 that the number of HCC with iso to high HBP signal intensity is small, and while statistically significant, may not be representative. However, the number of HCCs with iso to high HBP signal intensity and without nonrim arterial hyperenhancement is reported to be extremely rare (about 0.7%, 2/304) among HCCs (Choi JW, Lee JM, Kim SJ, Yoon JH, Baek JH, Han JK, et al. Hepatocellular carcinoma: imaging patterns on gadoxetic acid-enhanced MR Images and their value as an imaging biomarker. Radiology. 2013;267(3):776-86). This is partly because most HCC with iso to high HBP signal intensity are categorized as LR-3, LR-4 and LR-5 and are rarely categorized as LR-M. However, while there are only three patients in our study, considering that these patients are BCLC stage 0/A and Child Pugh class A, they can be possible candiates of liver transplantation. In such sense, we do not believe that three out of 106 LR-M (~3%) is a small number. However, we understand Reviewer #1’s concern and thus we made sure to “tone down” the significance of these findings and revised our sentences in both results section and discussion section.

**Comment #8** -“ Nearly half of 42 dark observations (22, 51%) were found to be while”. The word HCC is missing.

🡪 We are deeply sorry for this mistake, and we have revised the above sentence.

**Comment #9** - “Out of 106 LR-M observations, 42 observations (42%) were assigned dark, 61 observation (58%) were assigned low, and 3 observations (3%)” Percentages are incorrect: 42/106 (39.6%), 61/106 (57.54%), 2/106 (2.8%)

🡪 We are deeply sorry for this mistake and revised the above % from 42% to 40%.

**Comment #10** “In case of iso-to-high observations, all three observations were found to be HCC although this association was not found to be statistically significant (P=0.060) (S4 Table). “ Was the HBP phase adequate in these patients? I am pretty sure it was not adequate in one of this case as it is shown in Figure 2 and this is not an adequate HBP

🡪 We thank Reviewer #1 for his or her comment. As mentioned above, the HBP images were quality controlled in this retrospective study. In addition, Figure 3 instead of Figure 2 is an example of HBP iso to high LR-M observation and HBP was adequate in these patients (Figure 2 is an example of HBP dark and low group).

Tables

**Comment #11** Please check ALL the % in the tables

🡪 We have checked all the % in the tables and manuscript and revised accordingly.

**Discussion**

**Comment #12** - All the first sentence of the discussion needs to be deleted as in one of the case (Fig 2) HBP was inadequate and because these are only 3 cases and you cannot draw conclusions on this. I would highlight other interesting points.

🡪 We thank Reviewer #1 for his or her comment. As already mentioned above, HBP was adequately assessed in our study, and Figure 2 is not an example of HBP iso to high but rather HBP dark to low group, and while three cases were obtained for iso to high group, their associations with well differentiated HCC and pseudoglandular pattern were statistically significant (Table 4). Similar to comment mentioned in Results section, we, however, understand Reviewer’s concern and thus revised our sentences to “tone-down” the significance of results obtained for HBP iso to high LR-M observations.

**Comment #13** -multisetep—>multistep

🡪 We have made the above correction as suggested by Reviewer #1.

**Comment #14** -“Likewise, significant association between cHCC-CCA 275 or iCCA and low signal intensity in

hepatobiliary is consistent with previous studies (25-27). Such low signal intensity in

hepatobiliary phase is thought to relate to the presence of abundant stromal fibrosis in iCCA

and cholangiocarcinoma component of cHCC-CCA, which causes extracellular accumulation

of contrast agent through large interstitial spaces (26, 28)."

- It is not exactly this way. When you have this abundant stroma you may have a cloud appearance as shown in this paper: Insights Imaging. 2021 Jan 12;12(1):8. doi: 10.1186/s13244-020-00928-w.

🡪 We thank Reviewer #1 for his or her comment. The cloud appearance (also referred to as “EOB-cloud” in some studies) in which the central portion of the tumor shows delayed enhancement is the reason why we believe these tumors were grouped as “low” in HBP (lower than liver parenchyme enhancement but not as dark as those categorized as HBP “dark” group). Cloud appearance, as suggested by Reviewer #1 is due to stromal fibrosis, and in this paper (Jeong HT, Kim M-J, Chung YE, Choi JY, Park YN, Kim KW. Gadoxetate Disodium–Enhanced MRI of Mass-Forming Intrahepatic Cholangiocarcinomas: Imaging-Histologic Correlation. American Journal of Roentgenology. 2013;201(4):W603-W11), which performed an image-histology correlation, the authors state that “large interstitial spaces seen in stromal fibrosis may retain contrast agents… the target appearance is obtained… also with gadoxetate disodium (page W610)”. Based on this reference, we meant to state that tumors in HBP low group are categorized in this group due to the cloud appearance which may have a pathologic basis to the abovementioned retained contrast in interstitial spaces due to stromal fibrosis.

**Comment #15** - Conclusion must be rewritten without focusing on those 3 iso-to-high HBP lesions for the reasons mentioned above.

- As suggested by Reviewer #1, we excluded our results on HBP iso-to-high LR-M observations in conclusion and mentioned only about HBP low LR-M observations.
