# Supplementary material for: Hepatobiliary phase signal intensity: A potential method of diagnosing HCC with atypical imaging features among LR-M observations
Source: PLoS One. 2021 Sep 13;16(9):e0257308. doi: 10.1371/journal.pone.0257308 (PMC8437291; doi:10.1371/journal.pone.0257308)
Supplement: Attachment — Submitted filename: Response to Reviewers.docx [file pone.0257308.s007.docx]

Review Comments to the Author:

Please use the space provided to explain your answers to the questions above. You may also include additional comments for the author, including concerns about dual publication, research ethics, or publication ethics. (Please upload your review as an attachment if it exceeds 20,000 characters)

🡪 We foremost thank the Reviewer for his or her thoughtful comments and for reviewing our manuscript.

Reviewer #1: 1. In the abstract and in the main document please change “parenchyme“ to parenchyma
🡪 As recommended, we changed the word “parenchyme” to “parenchyma” in the abstract and main manuscript.

2. “Yes, we used 10mL of gadeoxetic-disodium regardless of patient’s weight as it is our hospital protocol ”
This needs to be added as a limitation because this is not what it is recommended in the drug leaflet

🡪 As recommended, we included this as a limitation in the discussion section.

3. Regarding figure 2 I understand that the contrast agent was in the bile ducts but the lower signal of vessels compared to liver parenchyma is also a criterion to judge adequacy of the HBP. I know that this feature may lack even after 40 mins after contrast injection typically in patients with high bilirubin levels, but Figure 2 and the lack of all criteria for HBP adequacy may raise concern from the reader’s perspective. I suggest to identify a different case for Figure 2, if this is not too much burden for you. In case this is highly difficult, please feel free to disregard this comment
🡪 We agree with the Reviewer that changing figure 2 example of low group would be better for the readers. As recommended, we modified Figure 2 using different case.

4. In figure 2 and 3 it is not needed to have an arrow for each figure part. Just leave it in the first image showing the lesion (2a, 2d and 3a) and delete it from the remaining.

🡪 As recommended, we removed arrows in Figure 2 and 3 and left it in first images (2a, 2d and 3a).
